# Malaria training for community health workers in the setting of elimination: a qualitative study from China

**DOI:** 10.1186/s12936-018-2229-1

**Published:** 2018-02-23

**Authors:** Guangyu Lu, Yaobao Liu, Jinsong Wang, Xiangming Li, Xing Liu, Claudia Beiersmann, Yu Feng, Jun Cao, Olaf Müller

**Affiliations:** 1 Medical College of Yangzhou University, Yangzhou University, Yangzhou, 225001 China; 2 Institute of Public Health, Medical School, The Ruprecht-Karls-Universität Heidelberg, INF 324, Bergheimerstraße 20, 69120 Heidelberg, Germany; 3 Key Laboratory of National Health and Family Planning Commission on Parasitic Disease Control and Prevention, Jiangsu Provincial Key Laboratory on Parasite and Vector Control Technology, Jiangsu Institute of Parasitic Diseases, Wuxi, China; 4 Gansu Provincial Center for Disease Control and Prevention, Lanzhou, China; 5 Public Health Research Center, Jiangnan University, Wuxi, China

**Keywords:** Malaria, Elimination, Training, Health workers, Perceptions, Expectations

## Abstract

**Background:**

Continuous training of health workers is a key intervention to maintain their good performance and keep their vigilance during malaria elimination programmes. However, countries progressing toward malaria elimination have a largely decreased malaria disease burden, less frequent exposure of health workers to malaria patients, and new challenges in the epidemiology of the remaining malaria cases. Moreover, competing health priorities and usually a decline in resources and in political commitment also pose challenges to the elimination programme. As a consequence, the acceptability, sustainability, and impact of malaria training and education programmes face challenges. However, little is known of the perceptions and expectations of malaria training and education programmes of health workers being engaged in countries with malaria elimination programmes.

**Methods:**

This qualitative study provides information on perceptions and expectations of health workers of malaria training programmes from China, which aims to malaria elimination by the year 2020. This study was embedded into a larger study on the challenges and lessons learned during the malaria surveillance strategy in China, involving 42 interviews with malaria experts, health staff, laboratory practitioners, and village doctors at the provincial, city, county, township, and village levels from Gansu province (northwestern China) and Jiangsu province (southeastern China).

**Results:**

In the context of an increasing number of imported malaria cases in China, the majority of respondents emphasized the necessity and importance of such programmes and complained about a decreasing frequency of training courses. Moreover, they called for innovative strategies to improve the implementation and sustainability of the malaria training programmes until the elimination goal has been achieved. Perceptions and expectations of health workers from different health centres were quite different. Health workers from higher-level facilities were more concerned about technical training aspects, while health workers from periphery of the health system expected to receive more training on field work coordination and on specific public health actions with regard to case detection and focus investigation.

**Conclusions:**

There is need to guarantee an ongoing good training of health workers in China on malaria aspects until the year 2020 and probably beyond.

## Background

Training and refresher training of health workers on malaria is of great importance and needs to continue in all countries as long as the disease persists in any part of the world [1]. Between 2000 and 2015, 17 countries eliminated malaria [2]. Currently, 35 countries are actively pursuing malaria elimination, with elimination goals ranging from 2016 to 2035 [3]. During the malaria elimination phase, malaria training for health workers is important to maintain vigilance among all health practitioners to ensuring well-functioning malaria surveillance [4]. However, countries progressing towards malaria elimination have a largely decreased disease burden and face to new challenges due to changes in malaria epidemiology, for example, indigenous malaria cases become less, while imported malaria cases increase. As a consequence, health workers face much less frequent exposure to malaria patients. In this scenario, strengthening malaria surveillance system play an important role in elimination programme, as well as training health workers to identify malaria cases. Consequently, the acceptability, sustainability, and the impact of malaria training and education programmes face major challenges [5–7].

China has achieved great success over the last 60 years in shrinking its malaria burden and has initiated its Malaria Elimination Programme in 2010, with the aim to have achieved this goal by 2020 [8]. Large-scale malaria training for health workers on aspects of microscopic diagnosis, vector control, epidemiology, treatment, and malaria prevention have played an important role in strengthening the primary health care system and have greatly contributed to the achievements of the Chinese malaria control programme [9–11]. For example, from 1979 to 1998, more than 700,000 health care providers were trained in epidemiology, entomology, parasitology, and malaria control [9]. In the transition from a malaria control programme to an elimination programme, the most significant change to the malaria epidemiology in China is the dramatic shrinking of indigenous malaria cases and the steady increase of imported malaria cases [12]. Therefore, it is important that health workers of different administrative levels remain vigilant in the detection of imported malaria cases to be able to manage such cases appropriately and to prevent further local transmission.

Health worker knowledge, attitudes, and practices on the diagnosis, treatment, and management of malaria have been evaluated in different epidemiological contexts, with the clear recommendation to provide malaria-related training programmes if such knowledge is not satisfactory [13–17]. However, little is known of the perceptions and expectations of malaria training and education programmes of health workers being engaged in countries with malaria elimination programmes. Therefore, this study investigated Chinese health workers’ perceptions on malaria training and education programmes during the ongoing nation Malaria Eradication Programme with the overall goal to strengthen the programme.

## Methods

This study is embedded into a larger qualitative study on “challenges and lessons learned during the implementation of the 1-3-7 surveillance strategy” [18].

### Study context

**Fig. 1:**
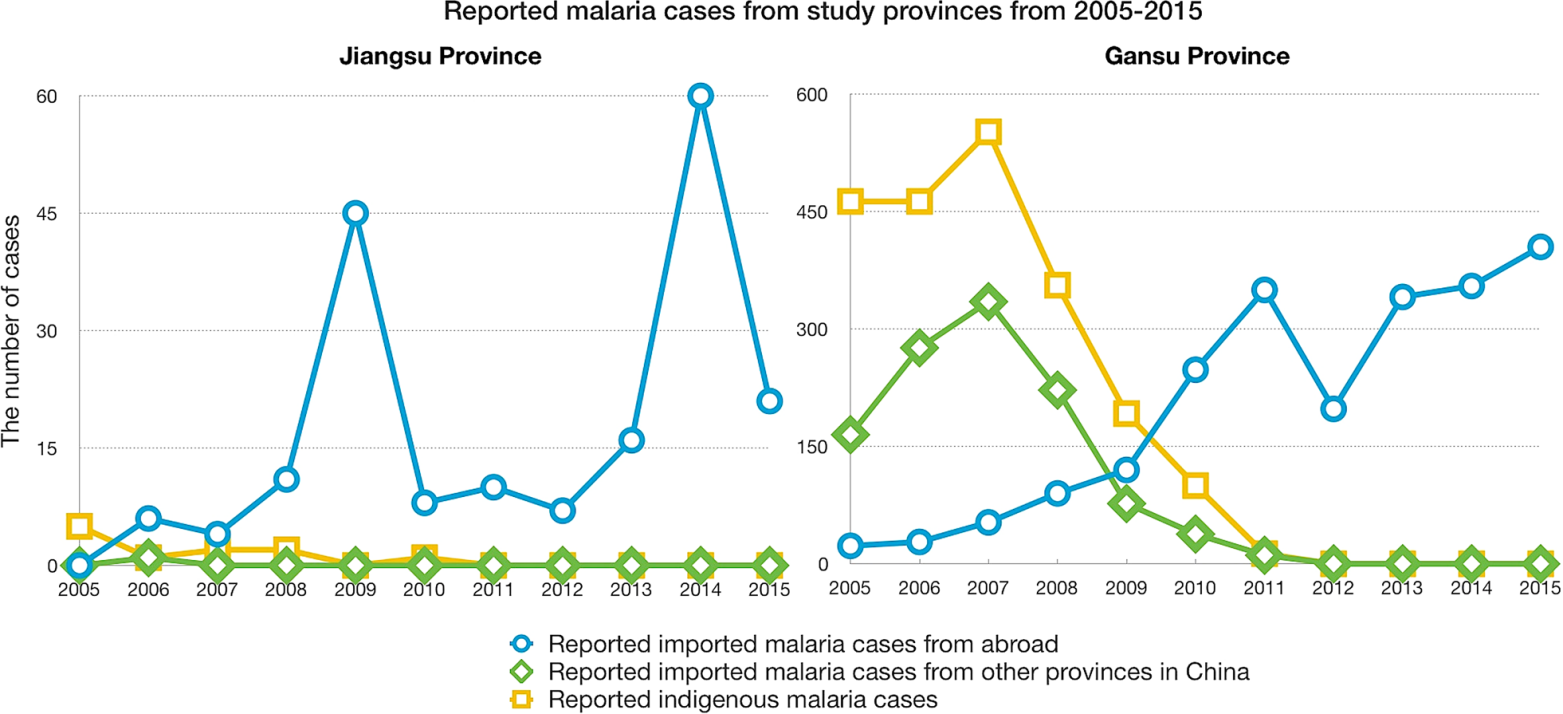
Reported malaria cases from study provinces from 2005 to 2015

The study was implemented in two provinces: Jiangsu province (southeastern China) and Gansu province (northwestern China) [18]. Jiangsu Province has been highly malaria endemic in the recent past, with about 10 million malaria cases reported each year during the 1960s and 1970s, where the annual incidence was as high as 250/1000 population [19]. Since 2012, there are no indigenous malaria cases reported from Jiangsu Province, but a continuous high number of imported malaria cases: in 2015, Jiangsu Province reported the second largest number of malaria cases (N = 405) amongst the 34 provinces/municipality/autonomous region of China, which all being imported malaria cases (Fig. 1).

Gansu Province was also malaria endemic in the past and its malaria burden decreased largely over the last decades. During the period of the year 2000 until the year 2010, less than 20 indigenous malaria cases were reported annually. However, also Gansu Province reported an increasing number imported malaria cases in recent years and a malaria epidemic with 60 imported malaria cases in 2014 in Wen County (20,000 inhabitants) (Fig. 1).

### Participants and sampling strategy

**Fig. 2:**
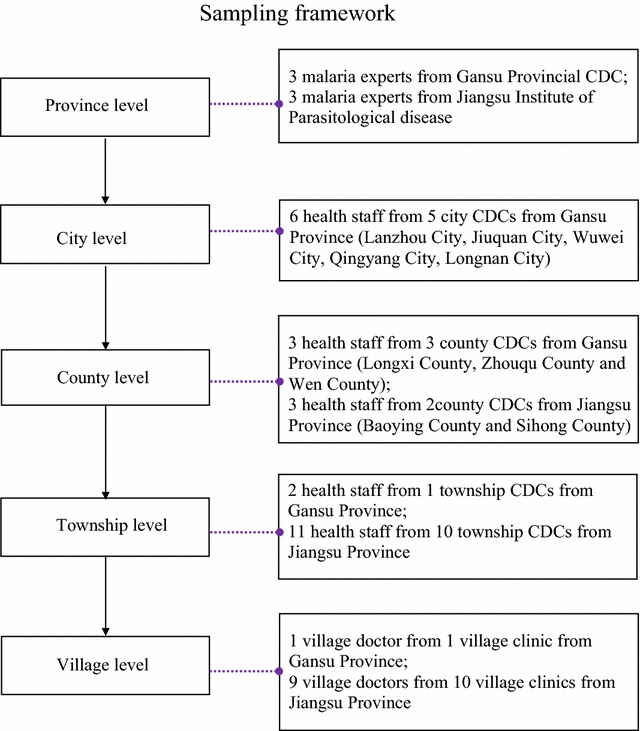
Sampling framework

The organizational structure of the provincial Chinese public health system is organized as follows: at the top are the provincial level departments, followed by the city Centers for Disease Control and Prevention (CDCs), and the county CDCs, and finally the township CDCs and village clinics in the periphery (Fig. 2) [18]. Before sampling started, a panel meeting including the main researcher (GL), malaria experts from provincial institutions or CDCs and health workers working from county level were organized. The sampling procedures have been described in detail elsewhere [18]. In short, the sampling procedures was a combing of purposive sampling and convenience sampling, based on epidemiological considerations, such as malaria prevalence, incidence and distribution of vectors, and specific information of local health staff (Fig. 2) [18]. Preference was given to areas with frequent reports of imported malaria cases since the initialization of the elimination programmes and with active vectors as transmission hotspots.

This study was designed to interview two groups of participants, namely (1) Chinese malaria experts and policy makers; and (2) health workers/epidemiologists involved into the routine malaria surveillance work at different levels of the health system. For the first group, almost all available malaria experts and policy makers from the provincial level were included. For the second group, intensity sampling (using information-rich cases) and snowball or chain sampling was used (utilizing well-informed people to identify critical cases or informants who have a great deal of information about a phenomenon) [18]. Information rich cases were considered as individuals who had participated in malaria case reporting, malaria epidemiological investigations, or specific malaria related public health actions over the last 3 years [18].

### Data collection

Two semi-structured questionnaires were designed: one for key informant interviews with malaria experts and policy-makers and the other for in-depth interviews with other health workers. A total of 42 respondents were interviewed for this study, including 6 malaria experts from Provincial Parasites Institute or Provincial CDCs, 6 health staff from the city CDCs, 6 health staff from county CDCs, 13 health staff from township hospital or township CDCs and 11 village doctors from village health centres. The data were collected during September and October of the year 2014.

The questions regarding malaria related training programmes were a separate section in the main questionnaire. Three main aspects on the topic were asked: (1) What do you think of current malaria training programme? (2) How frequent are the malaria training programmes conducted and how do you judge the current frequency? (3) What is the content of the present malaria training programmes and what are your expectations on future programmes?

### Data management and analysis

GL transcribed the interviews into Chinese. The transcribed transcripts were cross-checked by the YL. Coding process followed to identify the features of the data, based on the original Chinese transcripts [20]. The coding process was carried out by two authors (GL and YL), followed by cross checking of the data by one author (GL). Data analysis was conducted by adopting a combing of deductive coding (based on the questionnaires) and inductive coding (to ensure no codes were missed) facilitated by the software MAXQDA 12.1.1. Then the themes were identified and developed. The themes were related to the general perceptions on malaria training programmes, perceptions on the frequency of the malaria training programmes, and the expectations of the future training programmes. Coding, development, and refinement of the themes were done by two authors (GL and YL).

### Ethics statement

This study was approved by the local authorities of the study provinces and by the Ethical Committee of the Medical School at the Heidelberg University in Germany (S-363/2014). Detailed information on the purpose of the study, the duration of the interview and the reasons for tape-recording of the interview were explained to participants before the study. All participants were asked for their written informed consent before the interviews.

## Results

### Demographics of study population

The demographic characteristics and professional affiliations of the respondents were summarized in Table 1. In total 42 participants were interviewed for the study. One health worker had not received any specific training on malaria because he just started to work, and all others (97.7%, 41/42%) had received malaria related training programmes before.

**Table 1:** Demographic characteristics of participants

	Participants (N = 42)	
	*N*	(%)
Age		
20–29	6	14.3
30–39	15	35.7
40–49	14	33.3
≥ 50	7	16.7
Gender		
Male	32	76.2
Female	10	23.8
Working positions		
Provincial level	6	14.3
City level	6	14.3
County level	6	14.3
Township level	13	31
Village level	11	26.2

### Perceptions of malaria training programmes

Different perceptions of current malaria training programmes were identified here. The majority of respondents stated that the malaria training programmes are necessary and important even with the largely decreased malaria burden and much less frequent exposure to malaria cases in the elimination phase. Health workers from provincial, city, and county CDCs were more likely to provide supporting reasons for their argument on why they thought malaria training is necessary and important, while very few township health workers and village doctors did. The most frequently stated reason for the importance of training was the increasing number of imported malaria cases, attributed to population movements. The health workers were generally worried about being unfamiliar with the proper responses to the reemergence of malaria cases.

*“Malaria training programmes are necessary, because in our village there are always migrate workers returning from abroad every year, especially from the African countries, so it is important to have training on malaria.”* (#04 health worker from village level)

*“Whether we achieve the malaria elimination goal or not, we should have regular training… because there is the hidden danger of malaria transmission here. Skills and awareness need to be strengthened through training programmes.”* (#05 health worker from township level)

Skeptical opinions on the malaria training programmes among health workers were also identified, although only a few mentioned them. These perceptions included viewing malaria training as a form of extra work, malaria training programmes not making too much sense because there are presently no malaria patients, viewing the implementation of malaria training programmes as just a formality, and finding it is difficult to state whether training programmes are necessary, as there are no malaria patients. This skepticism on the necessity and importance of malaria training programmes was mainly identified in village and township health workers.

*“I think the annual malaria*-*related training programme is just a formality, which is a task for us to do.”* (#24 health worker from township level)

*“Now it is hard to say (whether the training is necessary or not). If we want to train someone (health workers or doctors), they will say that they are very busy, and there are no patients, so why do they need to be trained?”* (#25 health worker from village level)

### Frequency of malaria training programmes

The frequency of malaria training programmes differed by health administrative level. Therefore, the themes were identified according to the subcategories of (1) province; (2) city, county, township; and (3) village. Province-level health workers were more involved in providing malaria training and education. Malaria-related training was provided at least once every year to community workers in different CDCs and to hospital and clinical doctors. It was also mentioned that during the period of funding from the Global Fund to Fight AIDS, Tuberculosis, and Malaria, malaria training programmes were scheduled in advance and implemented more frequently. The provincial health workers identified the integration of malaria-related training programmes into the Provincial Continued Medical Education Programme (CME, a health education programme held every year; health workers from different CDCs participate regularly) as a promising strategy for sustaining malaria training programmes.

*“The malaria training was included into the provincial continued medical education programmes. So at least the training will be provided once a year.”* (#11 health worker from provincial level)

*“During the period of Global Fund period, I remember that we received training once a season, and now once a year.”* (#26 health worker from township level)

The respondents from city, county, and township CDCs reported consistently that they received malaria-related training once or twice a year from higher-level administrative health organizations. However, they reported that there was less frequent malaria training and education for community workers compared to previous years. Respondents mentioned that the frequency of the training programme depends greatly on the budget. Therefore, sustaining malaria training programmes by integrating them into a meeting/conference schedule is common.

*“The frequency of malaria training programmes depends on the budget. If there is not enough money, it is hard to provide training programmes.”* (#31 health worker from city level)

Village doctors reported that they received malaria-related training once or twice a year. The training is usually integrated into the regular meetings held by township CDCs, which is in a format termed “Meeting plus training”. The training is usually delivered during May to October. Village doctors generally considered the current frequency and means of the training on malaria acceptable because they are very busy and do not have time to specifically attend malaria training at the township CDCs. The health workers also noted that the malaria training was obviously less frequent than in previous years, the reason being the largely decreased number of malaria cases.

*“We are usually trained on malaria when we participate in the meeting at township CDCs. The meetings require all village doctors to participate, and we can get the malaria trainings through meetings, which is called Meeting plus Training.”* (#11 health worker from village level)

### Future expectations regarding training programmes

Health workers from different administrative levels play different roles in malaria elimination programmes. Therefore, the training content and expectations of future malaria training and education programmes were identified according to the subcategories of province, city, county, township, and village health administrative levels, respectively (Table 2).

**Table 2:** Themes of the future expectations regarding malaria training programs of health workers from different administrative health facilities

Different administrative levels of health facilities	Themes of expectations regarding malaria training programs
Province level	Hospital and clinic doctors should be included in malaria training programs Continued training of primary health care workers on malaria microscopic diagnostic skills is necessary
City level	Microscopic diagnosis skills training is important Training medical doctors on the knowledge of imported malaria is of great importance There is a transition of training programs from aspects of malaria control towards elimination in recent years
County level	Regular malaria microscopic diagnostic training regarded as very important A generally satisfaction with the content and organization of the current training program
Town level	Trained on basic aspects of malaria knowledge The training programs should be improved Current training focuses too much on malaria basics and there is insufficient attention on the guidelines and procedures of specific public health actions in the field More training on knowledge on the procedures of malaria-related public health actions in the field More information on how operational challenges during the implementation of malaria-related public health actions
Village level	Trained on basic knowledge including treatment of malaria More or only interested in knowledge on malaria prevention More willingness to be trained on how to suspect and detect malaria cases and the detailed procedures or specific activities during the focus investigation

### Province level

Provincial-level health workers believed that malaria training programmes should include hospital and clinic doctors to maintain the doctors’ vigilance in quickly detecting and diagnosing potential malaria cases. For example, health workers were previously trained to suspect and check for malaria among migrated workers returning from Africa or south Asia with classic malaria symptoms, whereas the current training for doctors is to be particularly alert for workers returning from Africa or south Asian with unexplained fever. Another important aspect emphasized by the province-level health workers was the necessity for continued training of primary health care workers on malaria microscopic diagnostic capacity.

*“We now train more and more clinical doctors, and tell them that if there are patients returning from Africa with unexplained fever, then they should consider checking for malaria Plasmodium. In the past, it was recommended that doctors check for malaria under the same conditions, but with suspected malaria symptoms.”* (#03 health worker from provincial level)

### City level

The health workers from the city CDCs also emphasized the importance of microscopic diagnosis capacity training. Moreover, they believed that training medical doctors on the knowledge of imported malaria is of great importance. The respondents generally noted that training programmes have gradually transitioned in recent years from focusing on aspects of malaria control towards aspects of elimination.

*“The medical doctors should be trained that if the patient has persistent fever and the fever does not abate, then you should ask about the travelling history of the patients and suspect malaria.”* (#32 health worker from city level)

*“In the recent 2* *years, the training was mainly focused on the guidelines and strategies.”* (#42 health worker from city level)

### County level

Health workers in county CDCs stated that, with the initiation of the malaria elimination programme, evaluation of training programme has increased. The county-level health workers receive regular malaria microscopic diagnostic training, and they believed that receiving training for this aspect is very important. The county CDCs health workers generally expressed satisfaction with the content and organization of the current training programme.

*“Usually at the beginning of the year, we will receive training on malaria policy, working requirements, and the evaluation method of malaria elimination programmes. At the same time, the microscopists receive regular training on the examination of Plasmodium.”* (#15 health worker from county level)

*“I think the current training is relatively good, as we could keep up*-*to*-*date on knowledge.”* (#18 health worker from county level)

### Town level

Town health workers reported that they mainly receive training on basic aspects of malaria knowledge. Some respondents felt that the training programmes should be improved. The town-level health workers believed that the current training focuses too much on malaria basics and there is insufficient attention on the guidelines and procedures of specific public health actions in the field. The other aspect identified was that malaria-related training should provide more information on how operational challenges during the implementation of malaria-related public health actions (e.g., indoor residue spraying at the focus) can be overcome in a coordinated way. Township CDC health workers generally reported an interest in knowledge on the procedures of malaria-related public health actions in the field, for example, protocols on how focus investigations following the identification of an imported malaria case during their work should be conducted.

*“I feel the training content is not suitable for our practical work. For example, we are required to visit malaria patients frequently, but it may be unrealistic to complete all these tasks in a limited time frame.”* (#37 health worker from township level)

*“The training is more on the malaria basic knowledge, but little on how to coordinate filed work. For example, training on how to conduct a focus investigation is not conducted in detail.”* (#05 health worker from township level)

### Village level

Village doctors believed that they receive training on the basic knowledge including treatment of malaria. However, the doctors shared that they were somehow more or only interested in knowledge on malaria prevention, as they would not be involved in the process of malaria treatment (The malaria treatment usually takes place in higher-level CDCs). Moreover, the village doctors were more willing to be trained on how to suspect and detect malaria cases and the detailed procedures or specific activities during the focus investigation.

*“I know we need to do spraying if a malaria case is identified in the village, but I do not know how to do it, especially in how many households I should do the spraying.”* (# 21 health worker from village level)

*“I think we need to be trained on how the focus investigation is to be conducted if a focus has been identified. As we do not treat the patients, we provide health education to the patients and households; therefore, knowledge on preventing malaria is important for us.”* (# 17 health worker from village level)

## Discussion

To our knowledge, this study is the first qualitative study to provide an understanding of the community health workers’ perceptions and their expectations of malaria training and education programmes in a malaria elimination setting globally. In general, the majority of respondents considered malaria training programmes necessary and important, although the disease burden is largely decreased and there is much less frequent exposure to cases in the malaria elimination setting. The respondents generally reported a satisfaction of the current malaria training programme, while, with the transition from control to elimination programmes, the decreasing frequency of malaria training programmes were concerned by the health workers. The findings also suggested that the perceptions and expectations of malaria training programmes of health workers from different administrative health centres are quite different. Health workers from higher-level administrative health centres were more concerned about receiving training on aspects of technique, while township CDCs and village clinic health workers expected to receive more training on field work coordination and the implementation of specific public health actions with regard to case detection and focus investigation.

Continued training of health workers during malaria elimination programmes, although acknowledged as being of great importance, faces various operational challenges with the decreasing disease burden, and often a curtailing of the resources and budgets [21]. Training and education are the traditional interventions for maintaining a good performance of health workers, which are certainly essential to malaria elimination programmes [22, 23]. The literature frequently reported assessments of the heath workers’ knowledge of malaria in different malaria endemic settings, and recommends more training if the results are not satisfactory [13–15, 17]. However, little is known of the perceptions and expectations of health workers, as training recipients, of malaria-related training programmes in a low malaria endemicity setting. The results generated in this study may thus also provide valuable information not only for Chinese health professionals for better planning, managing, and evaluation of malaria training programmes, but also to other countries in the malaria pre-elimination or elimination phase to wisely allocate resources.

### Perceptions of malaria training

The majority of health workers in our study considered malaria training programmes necessary and important even with the largely decreasing exposure to malaria patients. The most frequently reported reasons for the importance of the training programmes was the continually increasing number of imported malaria cases in recent years. In China, imported malaria cases have risen from 18.8% (1372/7312) of total reported malaria cases in 2010 to 98.8% (3248/3288) in 2015 [24, 25]. Many participants in this study acknowledged and considered this increase is the greatest threat to the malaria programme in China. In areas with a potential for establishing further on-going transmission (hotspots) in particular, the health workers emphasized the importance of regular training on malaria in order to remain familiar with the disease and to be prepared with proper responses to identified malaria cases.

However, this study also identified skeptical opinions on malaria training programmes, and the main doubt concerns the necessity of training programmes in case there are no patients. Therefore, trainers should prioritize education on the importance of the training on specific malaria knowledge or diagnostic skills to health workers. Moreover, it is important to notice that health workers from lower-level administrative health centres (i.e. township and village health centres) reported doubts on training programmes more frequently. This could probably be explained by previous research findings reporting that the formal education of healthcare providers is associated with malaria related knowledge, attitude and practice; in China, township or village health centre health workers usually have lower education levels compared to those from higher-level administrative health centres [26, 27]. This indicates that trainers should take into account the skeptical perceptions of health workers from middle or low administrative health centres, as these skepticisms on malaria training programme will reduce the training effects.

### Frequency of malaria training

The respondents generally reported that malaria training programmes regularly take place once or twice a year, while reflecting that the training was not as frequently in recent years and in particular compared to the period of funding through the Global Fund to Fight AIDS, Tuberculosis, and Malaria (2003–2012). Allocating limited resources wisely and sustaining proper training programmes require innovative strategies during the malaria elimination phase [23]. In this study, two innovative strategies for sustaining a regular frequency of training programmes were identified. The first is to integrate malaria-specific training programme into Provincial Continued Medical Education Programme for health workers, which regularly took place every year for health workers from different CDCs. The second is to organize training in “meeting plus training” formats, which combines training with specific conference or regular meetings. The latter form is generally well-received by the health workers especially village doctors. Other innovative, non-traditional training methods such as computer-based training might be less expensive, more flexible in time and places and promising, but requires substantial technology assistance [23].

### Expectations of malaria training

The health workers’ expectations of malaria training programmes differed largely based on administrative levels. Generally, the respondents reported positive views on the current malaria training programmes, while the findings suggested that training programmes for township health workers and village doctors require improvement. Specifically speaking, more information on how to detect malaria cases and conduct focus investigations were expected from the township health workers and village doctors. Moreover, this study observed a gap in the expectations of the training content between health workers from higher-level administrative health centres (e.g., provincial CDCs, usually the trainers or organizers) and the township CDC health workers/villages doctors. The province-level health workers emphasized the importance of being trained on technical aspects (e.g., microscopic diagnostic skills) while the township CDC health workers and village doctors expected more knowledge and guidance on detecting imported malaria cases, on field work coordination, and on specific procedures of public health actions after the identification of a case. Therefore, to optimize training programmes in the elimination phase, careful consideration should be given to the local epidemiology, demographic factors, tasks involved, and personal interests of the participants according to their roles and responsibilities. For examples, for doctors and health workers mainly working in the townships and villages, more knowledge could be provided on malaria case detection, field work coordination and procedures of focus investigation; for health workers working in the county or city CDCs, more training could be provided on malaria microscopic diagnostic skills.

With the programme goal transitioning from control to elimination, besides training health workers to be alert to imported malaria cases, microscopic diagnostic skills and prompt focus investigation capacity also need to be considered as important for inclusion in the training programmes. First, training on the prevention of malaria re-introduction is important, especially in the context of the increasing number of imported malaria cases [28–30]. Second, training on inter-sectoral and cross-border collaboration needs more emphasis. Explaining the role of the community and providing examples of successful inter-sector collaborations could further motivate health workers to address remaining challenges [31–33]. Finally, explaining the procedures for WHO certification of malaria elimination would enhance the health workers’ understanding of the reporting requirements and the evaluation indicators [31].

### Differences between different administrative health centres

Respondents from different administrative health centres have quite different perceptions and expectations of the malaria training programmes. The lower-level administrative centre health workers reported more doubts, more improvements, and less motivation for receiving malaria training. This could be because such health workers usually have lower education levels in China [34]. Moreover, it also reflects the fact that low-level administrative centre health workers such as village doctors, although serving the rural areas and poor, often receive insufficient remuneration or work incentives, along with insufficient continuing education and acknowledgement [34, 35]. However, given the great importance of village doctors and township CDC health workers in suspecting and detecting malaria cases and their important role in facilitating the implementation of public health actions, adequate and sustained motivation and training of low-level administrative centre health workers should be considered in future planning of malaria training programme [36]. For example, training the village doctors and township CDC health workers on timely referral suspected malaria patients to county CDCs is a viable option.

In countries approaching elimination goal, imported cases become increasingly significant and thus threaten re-establishment of malaria transmission in receptive areas. Therefore, countries toward malaria elimination should understand the changing malaria epidemiologic features and make proper strategies (such as establishing inter-sectorial collaborations and strengthening the surveillance system) to prevent the re-introduction of the imported malaria cases. More importantly, the knowledge on these changing epidemiological knowledge and specific procedures of the strategies should be provided to the health workers working in different administrative levels, and it will be essential for the implementation of the elimination programme. Considering the patterns of the imported malaria cases and the health systems of different countries are different, therefore, it would be important to understand the opinions of the health workers regarding malaria training programmes in different countries in order to maximize the impact of malaria training programmes.

### Strengths and limitations of the study

This study is the first qualitative investigation on community health workers’ perceptions and expectations regarding malaria training and education programmes during a malaria elimination phase. It can also be considered a strength of the study that all interviews were conducted by one researcher who had not worked for a Chinese authority at that time. This has likely decreased the pressure on the respondent to give politically correct and thus potentially biased answers towards the limitation of the current malaria training programmes. A limitation of the study is that the study areas are not representative for the whole of China. For example, the health staff working in cross-border provinces, may face special training needs on cross-border collaborations.

## Conclusions

China has made great achievements in malaria control and is now moving malaria elimination. Providing continued training to health workers to maintain good performance during the malaria elimination programme is important for rapid case detection, diagnosis, and treatment, and for interrupting the further potential transmission. The majority of health workers recognized well the necessity and importance of malaria training programmes, although there is a less frequent exposure of them to malaria cases in the ongoing national elimination programme. It is of great importance to notice that the perceptions and expectations of malaria training programmes of health workers from different administrative health centres are quite different. Therefore, to maximize the impact of malaria training programmes in malaria elimination settings, the perceptions and expectations of health workers from different administrative levels regarding malaria training programme need to be considered.
